# Supplementary material for: Sex-modulated association between thyroid stimulating hormone and informant-perceived anxiety in non-depressed older adults: Prediction models and relevant cutoff value
Source: Sci Rep. 2025 Jan 20;15:2526. doi: 10.1038/s41598-025-86703-7 (PMC11747398; doi:10.1038/s41598-025-86703-7)
Supplement: Supplementary file 1 — Supplementary Information. [file 41598_2025_86703_MOESM1_ESM.docx]

**Supplementary material**

**Sex-modulated association between thyroid stimulating hormone and informant-perceived anxiety in non-depressed older adults: Prediction models and relevant cutoff value**

Asma Hallab ^a,b,c,*^

**Affiliations**

a- Biologie Intégrative et Physiologie – Neurosciences Cellulaires et Intégrées. Faculté des Sciences et Ingénierie, Sorbonne Université, Paris, France.

b- Pathologies du sommeil. Hôpital Universitaire Pitié-Salpêtrière. Faculté de Médecine, Sorbonne Université, Paris, France.

c- Charité - Universitätsmedizin Berlin, Corporate member of Freie Universität Berlin and Humboldt-Universität zu Berlin. Berlin, Germany.

**^*^Corresponding author:** Dr. med. Dr. Asma Hallab. Charité Universitätsmedizin – Berlin. Charitéplatz 1, 10117 Berlin – Germany. [asma.hallab@charite.de](mailto:asma.hallab@charite.de).

https://orcid.org/0000-0002-3901-7980

**Supplementary Table 1:** Detailed models of the association between thyroid function (TSH continuous) and anxiety symptoms in the total study population and sex strata

|  | **Model 1** | | | | **Model 2** | | | | **Model 3** | | | | |
| --- | --- | --- | --- | --- | --- | --- | --- | --- | --- | --- | --- | --- | --- |
| **Characteristic** | **N** | **Event** | **OR** (**95% CI**) | ***p*-value** | **N** | **Event** | **OR** (**95% CI**) | ***p*-value** | **N** | **Event** | **OR** (**95% CI**) | ***p*-value** |  |
| **Total study population** | | | | | | | | | | | | | |
| **TSH (µIU/mL)** | 2,114 | 290 | 0.86 (0.76, 0.97) | **0.011** | 2,114 | 290 | 0.85 (0.75, 0.96) | **0.007** | 1,941 | 265 | 0.84 (0.73, 0.96) | **0.009** |  |
| **Sex** |  |  |  |  | 2,114 | 290 |  | 0.065 | 1,941 | 265 |  | 0.118 |  |
| Female |  |  |  |  |  |  | — |  |  |  | — |  |  |
| Male |  |  |  |  |  |  | 1.27 (0.99, 1.65) |  |  |  | 1.27 (0.94, 1.72) |  |  |
| **Age (years)** |  |  |  |  | 2,114 | 290 | 0.99 (0.97, 1.00) | 0.106 | 1,941 | 265 | 0.97 (0.95, 0.99) | **0.009** |  |
| **Racial profile** |  |  |  |  | 2,114 | 290 |  | **0.044** | 1,941 | 265 |  | 0.160 |  |
| White |  |  |  |  |  |  | — |  |  |  | — |  |  |
| Black |  |  |  |  |  |  | 0.51 (0.26, 0.91) |  |  |  | 0.51 (0.22, 1.05) |  |  |
| Other |  |  |  |  |  |  | 1.27 (0.70, 2.17) |  |  |  | 1.20 (0.55, 2.39) |  |  |
| **BMI** |  |  |  |  | 2,114 | 290 | 0.96 (0.93, 0.99) | **0.010** | 1,941 | 265 | 0.98 (0.95, 1.01) | 0.280 |  |
| **ADAS_13_ total score** |  |  |  |  |  |  |  |  | 1,941 | 265 | 1.02 (1.00, 1.04) | **0.031** |  |
| **Main diagnosis** |  |  |  |  |  |  |  |  | 1,941 | 265 |  | **<0.001** |  |
| HC |  |  |  |  |  |  |  |  |  |  | — |  |  |
| MCI |  |  |  |  |  |  |  |  |  |  | 3.40 (2.17, 5.51) |  |  |
| Dementia |  |  |  |  |  |  |  |  |  |  | 5.76 (3.11, 10.8) |  |  |
| **APOE ε4 alleles** |  |  |  |  |  |  |  |  | 1,941 | 265 | 1.17 (0.95, 1.43) | 0.139 |  |
| **Educational level (years)** |  |  |  |  |  |  |  |  | 1,941 | 265 | 0.94 (0.89, 0.99) | **0.012** |  |
| **Home** |  |  |  |  |  |  |  |  | 1,941 | 265 |  | 0.662 |  |
| House or apartment |  |  |  |  |  |  |  |  |  |  | — |  |  |
| Retirement or nursing institution |  |  |  |  |  |  |  |  |  |  | 0.75 (0.29, 1.66) |  |  |
| Other |  |  |  |  |  |  |  |  |  |  | 1.34 (0.47, 3.24) |  |  |
| **Retirement status** |  |  |  |  |  |  |  |  | 1,941 | 265 | 1.10 (0.77, 1.57) | 0.614 |  |
| **Marital status** |  |  |  |  |  |  |  |  | 1,941 | 265 | 1.37 (0.95, 1.95) | 0.089 |  |
| **Males** | | | | | | | | | | | | | |
| **TSH (µIU/mL)** | 1,117 | 166 | 0.71 (0.58, 0.85) | **<0.001** | 1,117 | 166 | 0.71 (0.59, 0.86) | **<0.001** | 1,039 | 153 | 0.71 (0.57, 0.86) | **<0.001** |  |
| **Age (years)** |  |  |  |  | 1,117 | 166 | 0.99 (0.96, 1.01) | 0.278 | 1,039 | 153 | 0.99 (0.96, 1.01) | 0.321 |  |
| **Racial profile** |  |  |  |  | 1,117 | 166 |  | 0.368 | 1,039 | 153 |  | 0.109 |  |
| White |  |  |  |  |  |  | — |  |  |  | — |  |  |
| Black |  |  |  |  |  |  | 0.67 (0.25, 1.50) |  |  |  | 0.61 (0.17, 1.70) |  |  |
| Other |  |  |  |  |  |  | 1.49 (0.68, 3.00) |  |  |  | 2.48 (0.95, 5.92) |  |  |
| **BMI** |  |  |  |  | 1,117 | 166 | 0.96 (0.91, 1.00) | **0.048** | 1,039 | 153 | 0.97 (0.92, 1.01) | 0.144 |  |
| **ADAS_13_ total score** |  |  |  |  |  |  |  |  | 1,039 | 153 | 1.01 (0.98, 1.04) | 0.408 |  |
| **Main diagnosis** |  |  |  |  |  |  |  |  | 1,039 | 153 |  | **<0.001** |  |
| HC |  |  |  |  |  |  |  |  |  |  | — |  |  |
| MCI |  |  |  |  |  |  |  |  |  |  | 4.12 (2.17, 8.53) |  |  |
| Dementia |  |  |  |  |  |  |  |  |  |  | 8.74 (3.73, 21.6) |  |  |
| **APOE ε4 alleles** |  |  |  |  |  |  |  |  | 1,039 | 153 | 1.12 (0.86, 1.47) | 0.402 |  |
| **Educational level (years)** |  |  |  |  |  |  |  |  | 1,039 | 153 | 0.96 (0.90, 1.03) | 0.221 |  |
| **Home** |  |  |  |  |  |  |  |  | 1,039 | 153 |  | 0.110 |  |
| House or apartment |  |  |  |  |  |  |  |  |  |  | — |  |  |
| Retirement or nursing institution |  |  |  |  |  |  |  |  |  |  | 0.18 (0.01, 0.91) |  |  |
| Other |  |  |  |  |  |  |  |  |  |  | 1.10 (0.16, 4.44) |  |  |
| **Retirement status** |  |  |  |  |  |  |  |  | 1,039 | 153 | 0.83 (0.50, 1.41) | 0.477 |  |
| **Marital status** |  |  |  |  |  |  |  |  | 1,039 | 153 | 1.41 (0.77, 2.46) | 0.256 |  |
| **Females** | | | | | | | | | | | | | |
| **TSH (µIU/dL)** | 997 | 124 | 1.01 (0.86, 1.18) | 0.878 | 997 | 124 | 0.99 (0.84, 1.16) | 0.934 | 902 | 112 | 0.96 (0.80, 1.14) | 0.674 |  |
| **Age (years)** |  |  |  |  | 997 | 124 | 0.99 (0.96, 1.01) | 0.253 | 902 | 112 | 0.96 (0.93, 0.99) | **0.014** |  |
| **Racial profile** |  |  |  |  | 997 | 124 |  | 0.099 | 902 | 112 |  | 0.113 |  |
| White |  |  |  |  |  |  | — |  |  |  | — |  |  |
| Black |  |  |  |  |  |  | 0.42 (0.16, 0.93) |  |  |  | 0.44 (0.13, 1.17) |  |  |
| Other |  |  |  |  |  |  | 0.98 (0.37, 2.22) |  |  |  | 0.39 (0.06, 1.40) |  |  |
| **BMI** |  |  |  |  | 997 | 124 | 0.97 (0.93, 1.01) | 0.118 | 902 | 112 | 1.00 (0.96, 1.05) | 0.906 |  |
| **ADAS_13_ total score** |  |  |  |  |  |  |  |  | 902 | 112 | 1.03 (1.00, 1.06) | **0.034** |  |
| **Main diagnosis** |  |  |  |  |  |  |  |  | 902 | 112 |  | **0.005** |  |
| HC |  |  |  |  |  |  |  |  |  |  | — |  |  |
| MCI |  |  |  |  |  |  |  |  |  |  | 2.73 (1.44, 5.38) |  |  |
| Dementia |  |  |  |  |  |  |  |  |  |  | 3.84 (1.51, 9.86) |  |  |
| **APOE ε4 alleles** |  |  |  |  |  |  |  |  | 902 | 112 | 1.27 (0.92, 1.74) | 0.151 |  |
| **Educational level (years)** |  |  |  |  |  |  |  |  | 902 | 112 | 0.90 (0.83, 0.98) | **0.011** |  |
| **Home** |  |  |  |  |  |  |  |  | 902 | 112 |  | 0.560 |  |
| House or apartment |  |  |  |  |  |  |  |  |  |  | — |  |  |
| Retirement or nursing institution |  |  |  |  |  |  |  |  |  |  | 1.69 (0.56, 4.48) |  |  |
| Other |  |  |  |  |  |  |  |  |  |  | 1.37 (0.36, 4.25) |  |  |
| **Retirement status** |  |  |  |  |  |  |  |  | 902 | 112 | 1.32 (0.82, 2.16) | 0.258 |  |
| **Marital status** |  |  |  |  |  |  |  |  | 902 | 112 | 1.37 (0.85, 2.20) | 0.196 |  |
| **ADAS_13_**: Alzheimer’s Disease Assessment Scale-13 items, **APOE:** Apolipoprotein-E, **BMI**: Body-Mass Index, **CI:** Confidence Interval, **HC:** Healthy Controls, **MCI**: Mild Cognitive Impairment, **OR:** Odds Ratio.  **Model 1**: non adjusted model, **Model 2**: adjusted for age (years), sex (only in total population) + racial profile (“white”, “black”, “other”) + BMI (weight(kg)/height(m)^2^), **Model 3**: Model 2 + ADAS_13_ total score + main cognition-related diagnosis (“HC”, “MCI”, “Dementia”) + APOE ε4 alleles (number of alleles) + educational level (years) + home (“house or apartment”, “retirement or nursing institution”, “other”) + retirement status (binary) + marital status (“currently married” vs. “currently not married or unknown”). | | | | | | | | | | | | |  |

**Supplementary Table 2:** Detailed models of the association between thyroid function (TSH categorical) and anxiety symptoms in the total study population and sex strata

|  | **Model 1** | | | | **Model 2** | | | | **Model 3** | | | | |
| --- | --- | --- | --- | --- | --- | --- | --- | --- | --- | --- | --- | --- | --- |
| **Characteristic** | **N** | **Event** | **OR (95% CI)** | ***p*-value** | **N** | **Event** | **OR (95% CI)** | ***p-*value** | **N** | **Event** | **OR (95% CI)** | ***p-*value** |  |
| **Total study population** | | | | | | | | | | | | | |
| **TSH (µIU/mL)** | 2,114 | 290 |  | **0.048** | 2,114 | 290 |  | **0.039** | 1,941 | 265 |  | **0.021** |  |
| TSH > 2.4 |  |  | — |  |  |  | — |  |  |  | — |  |  |
| TSH ≤ 2.4 |  |  | 1.35 (1.00, 1.85) |  |  |  | 1.37 (1.02, 1.88) |  |  |  | 1.48 (1.06, 2.10) |  |  |
| **Sex** |  |  |  |  | 2,114 | 290 |  | 0.072 | 1,941 | 265 |  | 0.111 |  |
| Female |  |  |  |  |  |  | — |  |  |  | — |  |  |
| Male |  |  |  |  |  |  | 1.27 (0.98, 1.64) |  |  |  | 1.18 (0.95, 1.73) |  |  |
| **Age (years)** |  |  |  |  | 2,114 | 290 | 0.99 (0.97, 1.00) | 0.093 | 1,941 | 265 | 0.97 (0.95, 0.99) | **0.006** |  |
| **Racial profile** |  |  |  |  | 2,114 | 290 |  | 0.053 | 1,941 | 265 |  | 0.168 |  |
| White |  |  |  |  |  |  | — |  |  |  | — |  |  |
| Black |  |  |  |  |  |  | 0.52 (0.27, 0.93) |  |  |  | 0.51 (0.22, 1.05) |  |  |
| Other |  |  |  |  |  |  | 1.25 (0.69, 2.14) |  |  |  | 1.18 (0.54, 2.36) |  |  |
| **BMI** |  |  |  |  | 2,114 | 290 | 0.96 (0.93, 0.99) | **0.009** | 1,941 | 265 | 0.98 (0.95, 1.01) | 0.256 |  |
| **ADAS_13_ total score** |  |  |  |  |  |  |  |  | 1,941 | 265 | 1.02 (1.00, 1.04) | **0.026** |  |
| **Main diagnosis** |  |  |  |  |  |  |  |  | 1,941 | 265 |  | **<0.001** |  |
| HC |  |  |  |  |  |  |  |  |  |  | — |  |  |
| MCI |  |  |  |  |  |  |  |  |  |  | 3.42 (2.18, 5.53) |  |  |
| Dementia |  |  |  |  |  |  |  |  |  |  | 5.76 (3.11, 10.8) |  |  |
| **APOE ε4 alleles** |  |  |  |  |  |  |  |  | 1,941 | 265 | 1.16 (0.95, 1.42) | 0.144 |  |
| **Educational level (years)** |  |  |  |  |  |  |  |  | 1,941 | 265 | 0.94 (0.89, 0.99) | **0.012** |  |
| **Home** |  |  |  |  |  |  |  |  | 1,941 | 265 |  | 0.687 |  |
| House or apartment |  |  |  |  |  |  |  |  |  |  | — |  |  |
| Retirement or nursing institution |  |  |  |  |  |  |  |  |  |  | 0.76 (0.30, 1.69) |  |  |
| Other |  |  |  |  |  |  |  |  |  |  | 1.32 (0.47, 3.20) |  |  |
| **Retirement status** |  |  |  |  |  |  |  |  | 1,941 | 265 | 1.11 (0.78, 1.59) | 0.565 |  |
| **Marital status** |  |  |  |  |  |  |  |  | 1,941 | 265 | 1.38 (0.96, 1.97) | 0.079 |  |
| **Males** | | | | | | | | | | | | | |
| **TSH (µIU/mL)** | 1,117 | 166 |  | **<0.001** | 1,117 | 166 |  | **<0.001** | 1,039 | 153 |  | **<0.001** |  |
| TSH > 2.4 |  |  | — |  |  |  | — |  |  |  | — |  |  |
| TSH ≤ 2.4 |  |  | 2.28 (1.45, 3.75) |  |  |  | 2.28 (1.45, 3.76) |  |  |  | 2.50 (1.52, 4.35) |  |  |
| **Age (years)** |  |  |  |  | 1,117 | 166 | 0.99 (0.96, 1.01) | 0.227 | 1,039 | 153 | 0.98 (0.96, 1.01) | 0.201 |  |
| **Racial profile** |  |  |  |  | 1,117 | 166 |  | 0.411 | 1,039 | 153 |  | 0.131 |  |
| White |  |  |  |  |  |  | — |  |  |  | — |  |  |
| Black |  |  |  |  |  |  | 0.66 (0.25, 1.48) |  |  |  | 0.58 (0.16, 1.61) |  |  |
| Other |  |  |  |  |  |  | 1.39 (0.63, 2.81) |  |  |  | 2.27 (0.88, 5.42) |  |  |
| **BMI** |  |  |  |  | 1,117 | 166 | 0.95 (0.91, 1.00) | **0.037** | 1,039 | 153 | 0.96 (0.92, 1.01) | 0.127 |  |
| **ADAS_13_ total score** |  |  |  |  |  |  |  |  | 1,039 | 153 | 1.02 (0.99, 1.04) | 0.288 |  |
| **Main diagnosis** |  |  |  |  |  |  |  |  | 1,039 | 153 |  | **<0.001** |  |
| HC |  |  |  |  |  |  |  |  |  |  | — |  |  |
| MCI |  |  |  |  |  |  |  |  |  |  | 4.11 (2.16, 8.51) |  |  |
| Dementia |  |  |  |  |  |  |  |  |  |  | 8.61 (3.69, 21.2) |  |  |
| **APOE ε4 alleles** |  |  |  |  |  |  |  |  | 1,039 | 153 | 1.12 (0.86, 1.47) | 0.395 |  |
| **Educational level (years)** |  |  |  |  |  |  |  |  | 1,039 | 153 | 0.96 (0.90, 1.03) | 0.220 |  |
| **Home** |  |  |  |  |  |  |  |  | 1,039 | 153 |  | 0.158 |  |
| House or apartment |  |  |  |  |  |  |  |  |  |  | — |  |  |
| Retirement or nursing institution |  |  |  |  |  |  |  |  |  |  | 0.20 (0.01, 1.03) |  |  |
| Other |  |  |  |  |  |  |  |  |  |  | 1.07 (0.16, 4.30) |  |  |
| **Retirement status** |  |  |  |  |  |  |  |  | 1,039 | 153 | 0.87 (0.53, 1.49) | 0.613 |  |
| **Marital status** |  |  |  |  |  |  |  |  |  |  | 1.41 (0.78, 2.47) | 0.250 |  |
| **Females** | | | | | | | | | | | | | |
| **TSH (µIU/dL)** | 997 | 124 |  | 0.301 | 997 | 124 |  | 0.395 | 902 | 112 |  | 0.436 |  |
| TSH > 2.4 |  |  | — |  |  |  | — |  |  |  | — |  |  |
| TSH ≤ 2.4 |  |  | 0.80 (0.53, 1.22) |  |  |  | 0.83 (0.55, 1.28) |  |  |  | 0.83 (0.52, 1.35) |  |  |
| **Age (years)** |  |  |  |  | 997 | 124 | 0.98 (0.96, 1.01) | 0.237 | 902 | 112 | 0.96 (0.93, 0.99) | **0.014** |  |
| **Racial profile** |  |  |  |  | 997 | 124 |  | 0.121 | 902 | 112 |  | 0.136 |  |
| White |  |  |  |  |  |  | — |  |  |  | — |  |  |
| Black |  |  |  |  |  |  | 0.44 (0.17, 0.97) |  |  |  | 0.46 (0.13, 1.24) |  |  |
| Other |  |  |  |  |  |  | 0.97 (0.36, 2.20) |  |  |  | 0.39 (0.06, 1.40) |  |  |
| **BMI** |  |  |  |  | 997 | 124 | 0.97 (0.93, 1.01) | 0.115 | 902 | 112 | 1.00 (0.96, 1.04) | 0.966 |  |
| **ADAS_13_ total score** |  |  |  |  |  |  |  |  | 902 | 112 | 1.03 (1.00, 1.06) | **0.042** |  |
| **Main diagnosis** |  |  |  |  |  |  |  |  | 902 | 112 |  | **0.004** |  |
| HC |  |  |  |  |  |  |  |  |  |  | — |  |  |
| MCI |  |  |  |  |  |  |  |  |  |  | 2.77 (1.47, 5.46) |  |  |
| Dementia |  |  |  |  |  |  |  |  |  |  | 3.94 (1.56, 10.1) |  |  |
| **APOE ε4 alleles** |  |  |  |  |  |  |  |  | 902 | 112 | 1.28 (0.92, 1.76) | 0.137 |  |
| **Educational level (years)** |  |  |  |  |  |  |  |  | 902 | 112 | 0.90 (0.83, 0.97) | **0.010** |  |
| **Home** |  |  |  |  |  |  |  |  | 902 | 112 |  | 0.589 |  |
| House or apartment |  |  |  |  |  |  |  |  |  |  | — |  |  |
| Retirement or nursing institution |  |  |  |  |  |  |  |  |  |  | 1.68 (0.56, 4.44) |  |  |
| Other |  |  |  |  |  |  |  |  |  |  | 1.29 (0.33, 4.00) |  |  |
| **Retirement status** |  |  |  |  |  |  |  |  | 902 | 112 | 1.31 (0.82, 2.15) | 0.264 |  |
| **Marital status** |  |  |  |  |  |  |  |  | 902 | 112 | 1.41 (0.87, 2.26) | 0.162 |  |
| **ADAS_13_**: Alzheimer’s Disease Assessment Scale-13 items, **APOE:** Apolipoprotein-E, **BMI:** Body-Mass Index, **CI:** Confidence Interval, **MCI:** Mild Cognitive Impairment, **OR:** Odds Ratio, **TSH:** Thyroid Stimulating Hormone.  **Model 1**: non adjusted model, **Model 2**: adjusted for age (years), sex (only in total population) + racial profile (“white”, “black”, “other”) + BMI (weight(kg)/height(m)^2^), **Model 3**: Model 2 + ADAS_13_ total score + main cognition-related diagnosis (HC, MCI, Dementia) + APOE ε4 alleles + educational level (years) + home (“house or apartment”, “retirement or nursing institution”, “other”) + retirement status (binary) + marital status (“currently married” vs. “currently not married or unknown”) | | | | | | | | | | | | |  |
